# Development of a Single-Drop Microextraction with Derivatization Procedure for Analysis of Volatile Fatty Acids in Water Samples

**DOI:** 10.1007/s10337-017-3316-0

**Published:** 2017-05-25

**Authors:** Grażyna Wejnerowska

**Affiliations:** Department of Food Analytics and Environmental Protection, Faculty of Chemical Technology and Engineering, University of Science and Technology in Bydgoszcz, Seminaryjna 3 St., 85-326 Bydgoszcz, Poland

**Keywords:** Derivatization, *N*-Methyl-*N*-(*tert*-butyldimethylsilyl)trifluoroacetamide, Single-drop microextraction, Volatile fatty acids, Water analysis

## Abstract

A single-drop microextraction (SDME) was developed for the analysis of volatile fatty acids (VFAs) (C_2_–C_7_) in water by gas chromatography (GC) with flame ionization detection. The significant parameters affecting the SDME performance such as selection of microextraction solvent, extraction time, stirring rate, sample pH and temperature, and ionic strength were studied and optimized. To lower limits of detection, derivatization of VFAs by *N*-methyl-*N*-(*tert*-butyldimethylsilyl)trifluoroacetamide (MTBSTFA) was performed. The method developed requires very short time of extraction and derivatization (13 min) and it is characterized by a good precision (max RSD = 11.4%), linearity and relatively low limits of detection (from 8.3 mg L^−1^ for acetic acid to 0.008 mg L^−1^ for heptanoic acid). The results of the SDME in combination with GC show promising potential for the analysis of VFAs in water samples.

## Introduction

Volatile fatty acids (VFAs) are low-molecular-mass organic acids with a strong hydrophilic character. They originate from aerobic biodegradation of carbohydrates, proteins, and fats. Therefore, they are widely present in activated sludge, waste and landfill leachates, and wastewaters. Recently, the determination of VFAs became of increasing interest, since it has been found that they are involved in different processes, i.e., the biological removal of phosphorus from waters or nitrification–denitrification in activated sludge [1].

However, free fatty acids, especially the short-chain ones (C_2_–C_5_), are strongly hydrophilic compounds, and the conventional methods (e.g., LLE and SPE) often have poor efficiency and analytical precision. An alternative preconcentration method for sample preparation, the technique of solid-phase microextraction (SPME) has been applied to the determination of a large variety of volatile and semi-volatile analytes in several types of environmental matrices [2–4]. SPME has been applied for extraction of volatile fatty acids from water [1], sludge [5], air [5], cheese [6], and biological samples [7]. However, compounds containing polar groups in their structures should be derivatized to improve the quality of the GC separations. For SPME-GC of polar compounds, such as acids, derivatization can be performed in the aqueous sample or in the SPME fibre after the concentration step [5, 8].

In recent years, the attractive technique of single-drop microextraction (SDME) has been developed as an alternative to SPME, which is based on the miniaturization of the traditional liquid–liquid extraction method, by greatly reducing the solvent to aqueous ratio [3]. Similar to SPME, a microdrop of a water-immiscible solvent is left suspended on the tip of a conventional microsyringe, immersed in a water sample (DI-SDME) or a microsyringe located in the headspace of a sample [9]. During the process, the analytes migrate from liquid sample to organic solvent, and dissolve within. After a suitable exposure time, the solvent drop is retracted into the syringe and is then injected into the GC [9]. SDME avoids some problems of the SPME method, such as sample carry-over and fibre degradation; it is also fast and inexpensive and uses very simple equipment [9–13].

In this paper, single-drop microextraction of volatile fatty acids from aqueous samples and GC-FID detection was studied. Moreover, the results of studies on possibility to apply derivatization reaction to lower limits of detection have been presented. *N*-methyl-*N*-(*tert*-butyldimethylsilyl)trifluoroacetamide (MTBSTFA) was selected as the derivatization reagent.

## Materials and Methods

### Reagents and Chemicals

Acetic-C_2_ (≥99.5%), propionic-C_3_ (99.5%), butyric-C_4_ (≥99.5%), valeric-C_5_ (≥99%), caproic-C_6_ (≥99.5%), heptanoic-C_7_ (≥97%) acids, and linoleic acid methyl ester (≥99%) were obtained from Sigma-Aldrich (Steinheim, Germany). Isooctane (≥99.5%) was obtained from Fluka and carbon tetrachloride (>99.8%), *n*-hexane (for gas chromatography) and toluene (≥99.8%) was purchased from Merck (Darmstadt, Germany). Sodium chloride was used to adjust the ionic strength of the aqueous samples and the hydrochloric acid used to modify the sample pH was from POCh (Gliwice, Poland).

The standard stock solutions of acid compounds were prepared by dissolving 31.5 mg of C_2_, 29.8 mg of C_3_, and 24.0 mg of C_4,_ and 23.0.4 mg of C_5_, 18.15 mg C_6,_ and 17.05 mg C_7_ in 100 mL of deionised water and were stored at 4 °C in the darkness. Carbon tetrachloride was used as a solvent and linoleic acid methyl ester as an internal standard (0.5 µL of linoleic acid methyl ester in 10 mL of carbon tetrachloride).

*N*-methyl-*N*-(*tert*-butyldimethylsilyl)trifluoroacetamide (MTBSTFA) (≥97.0%) from Fluka was used as derivatization reagent (diluted in carbon tetrachloride 1:4). Optimization of DI-SDME technique with derivatization of VFAs was performed by the use of acid solutions at concentrations of: 12.59 mg of C_2_, 11.90 mg of C_3_, 9.58 mg of C_4_, 9.38 mg of C_5_, 7.26 mg of C_6,_ and 6.82 mg of C_7_ in 100 mL of deionised water.

### Procedure

#### Direct Single-drop Microextraction

The extraction and injection procedures were carried out using a Hamilton 10-µL GC microsyringe. A water sample spiked with an appropriate amount of VFA compounds and 0.2 g NaCl was introduced into an 8 mL glass vial (Supelco, Bellefonte, PA, USA) equipped with a screw cap and a silicone septum. The completely filled up vials were stirred by magnetic stirrer (500 rpm) at constant temperature. The plunger was depressed and the 2.0 µL drop of the organic phase (carbon tetrachloride) with internal standard (linoleic acid methyl ester) was immersed into the aqueous solution at room temperature for 12 min. After extraction, solvent was retracted into the microsyringe and transferred to the heated injection port of the gas chromatograph. Finally, the analytical signal was shown as the relative peak area of the analyte to the internal standard.

### Derivatization Procedure

After extraction (DI-SDME) of analytes to the drop (2.0 µL) of carbon tetrachloride, the drop was retracted and injected into an analytic vial of 0.3 mL. Then, MTBSTFA solution in carbon tetrachloride (1:4) was added in amount of 0.5 µL (for 1 min). Finally, the content of vial was reintroduced into microsyringe and GC-FID analysis was performed.

### Apparatus

All GC analyses were performed on an HP 5890 (Hewlett-Packard, USA) model equipped with a flame ionization detector (FID). Injection was into a split-splitless injector operated in the splitless mode. The fused silica capillary column coated with a 1.0 µm film of HP-FFAP (polyethylene glycol), 30 m and 0.53 mm i.d., was used. Helium was used as a carrier gas at a constant flow rate of 1.2 mL min^−1^. The temperature of the injector and the detector was 250 °C. The oven temperature was 70 °C for 2 min, and then programmed to 230 °C at 10 °C/min and held there for 2 min. In the case, when analyses were performed after derivatization, the oven temperature was 50 °C for 4 min, and then programmed to 130 °C at 8 °C/min, next then programmed to 220 °C at 20 °C/min.

## Results and Discussion

### Optimization of the Direct SDME

**Fig. 1:**
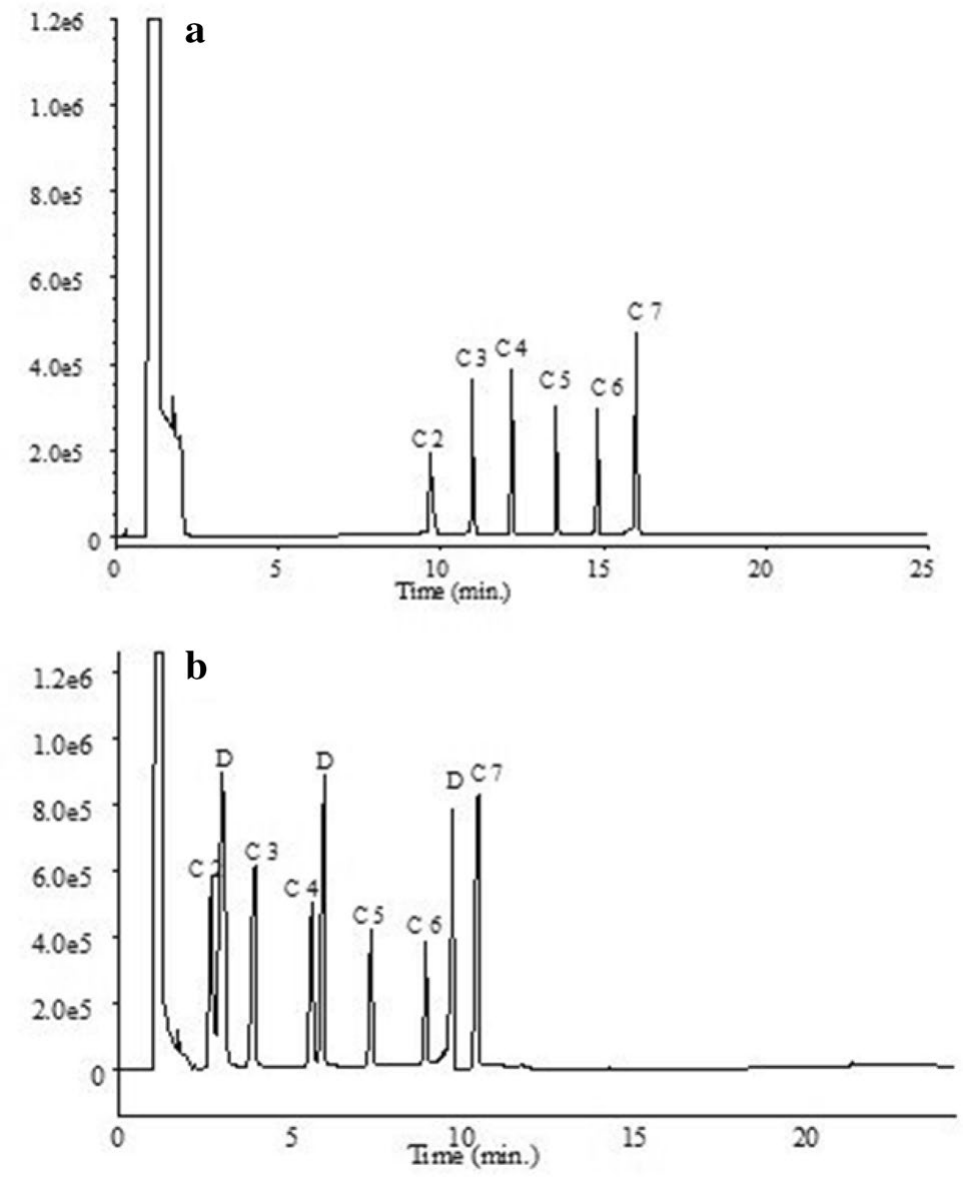
GC-FID chromatograms obtained by SDME: **a** DI-SDME, **b** DI-SDME with derivatization. D-derivatization reagent

The first optimization step for DI-SDME method is the selection of an appropriate extraction solvent and internal standard. Linoleic acid methyl ester was used as internal standard to correct for variation in injection volumes. Three water-immiscible solvents (*n*-hexane, carbon tetrachloride, and toluene) were tested to select the best one for the extraction of VFAs in water samples with this technique. The experiments were performed by applying extractions for 15 min at room temperature and stirring at 500 rpm, in completely filled vials of 8 mL. The highest efficiency of extraction was obtained for the first three acids (C_2_–C_4_) in carbon tetrachloride and for the other three in toluene. For the reason that FID detector shows lower sensitivity for the first three acids, carbon tetrachloride was selected as the extractant solvent (Fig. 1). It was also important that this solvent loss occurred very seldom during extraction.

The optimum extraction time was determined varying the time of exposing microdrop in the standard aqueous solution (from 5 to 18 min). The highest efficiency of extraction for all analytes was obtained after 12 min of extraction. Further prolongation of the extraction time resulted in decrease in efficiency of extraction for C_2_–C_4_ acids. An optimum sample extraction time of 12 min was, therefore, chosen for further studies.

**Fig. 2:**
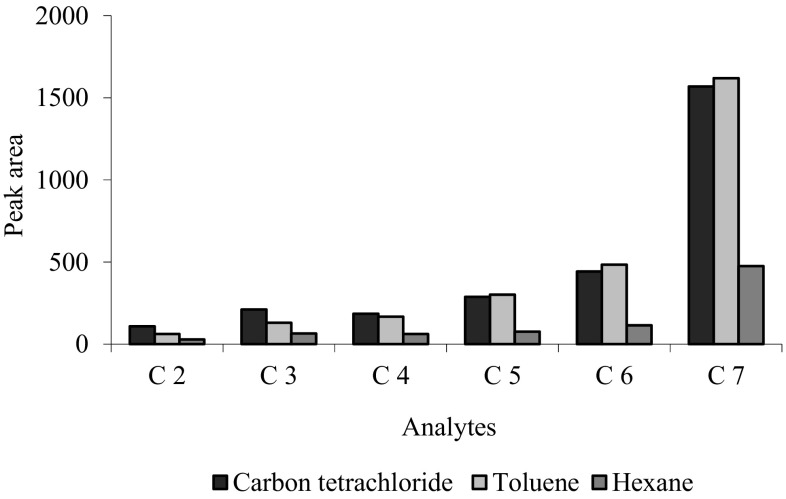
Effect of different organic solvents on the extraction efficiency for DI-SDME method. Concentration of analytes: 85–155 mg L^−1^, solvent volume: 2.5 µL, solution temperature: 22 °C, time extraction: 15 min, salt addition: 4% NaCl, stirring rate 500 rpm

The amount of extracted analyte depends on the microdrop volume. As shown in Fig. 2, the extraction efficiency enhances by increasing the microdrop volume up to 2.0 µL. Decrease in extraction efficiency was observed for microdrop volume of 2.5 µL (with the exception of C_7_). Drop volumes of 2.0 µL were considered in further experiments.

Sample stirring increases extraction efficiencies and reduces extraction time. Stirring rates above 500 rpm were not evaluated, because they made the microdrop unstable. Therefore, the optimum stirring rate was selected at 500 rpm and was used in all subsequent experiments.

The effect of temperature was studied by exposing a solvent drop in water samples at 22, 25, 30, and 35 °C. Experimental results showed that, by increasing the temperature, extraction efficiency decreased and that was why the further experiments were performed at room temperature.

The effect of increasing the ionic strength of the water sample was evaluated by adding NaCl in amounts from 0 to 12% (w/v). Experimental results showed that VFA peak areas increased even by about 100% when NaCl amounts were increased (the highest increase was observed for C_5_ and C_6_). However, because of the often occurring drop losses, the salt was not added to the samples studied in our further studies. Moreover, effect of pH reduction on extraction efficiency within the pH range from 2 to 5.8 was studied. The highest increase in signal (approx. 20%) was observed when pH of sample was equal to 3. However, likewise as in the case, when NaCl was added, the drop losses occurred very often, and therefore, HCl was not added to the samples in further experiments.

On the basis of the optimization method, the following conditions were chosen for the DI-SDME technique: 2.0 µL carbon tetrachloride microdrop, 500 rpm stirring rate, and 12 min extraction time and room temperature.

The next step of our studies comprised evaluation of DI-SDME suitability for the quantitative determinations of VFAs in water samples. The precision, linearity, and limits of detection were determined for each acid. The precision of the proposed DI-SDME techniques was determined by performing the extraction seven times. The relative standard deviations are shown in Table 1 and were in the range 8.0–11.0%.

**Table 1:** Comparison of the methods developed and results: linearity, precision, and limit of detection

Compound	DI-SDME				DI-SDME with derivatization				
	Linear range (mg L^−1^)	*R* ^2^	RSD (%) (*n* = 7)	LOD (mg L^−1^)	Linear range (mg L^−1^)	*R* ^2^	RSD (%) (*n* = 6)	LOD (mg L^−1^)	Enrichment factor
Acetic	150–310	0.982	9.7	18.2	40–126	0.980	7.2	8.3	132
Propionic	60–300	0.988	8.6	4.3	10–120	0.979	9.8	1.2	148
Butyric	20–240	0.962	11.0	2.1	8–96	0.967	11.2	0.83	98
Valeric	10–235	0.990	8.1	0.9	8–94	0.979	10.8	0.62	101
Caproic	1.2–185	0.989	8.0	0.09	0.2–72	0.978	11.4	0.01	96
Heptanoic	0.6–170	0.986	10.7	0.03	0.1–68	0.988	9.0	0.008	92

The linearity range for the individual acids (Table 1) were determined and correlation coefficient *R* ^2^ was higher than 0.962 (for C_4_). In general, the acids have shown good linearity along the whole evaluated range. As the length of acid chain is longer, LOD significantly decreases from 18.2 for C2 to 0.03 mg L^−1^ for C7. The limits of detection obtained by the methods developed were on a high level. Ábalos et al. [1], by applying headspace SPME-GC-FID method, obtained the limits of detection for acetic acid on the level 0.675 mg L^−1^ and the lowest ones for heptanoic acid on the level of 0.038 mg L^−1^. These limits are considerably lower than the limits obtained by DI-SDME method; a comparable LOD level was obtained only in the case of the less volatile acid (heptanoic acid). It probably results from the fact that the less volatile acid (heptanoic acid) shows higher efficiency of extraction in water phase than in headspace.

### Derivatization

Derivatization of fatty acids to their derivatives results in a change of physicochemical properties of the compounds under the study. Usually, volatility or polarity of analytes is changed which improves efficiency of extraction or separation of analytes on chromatographic column. Data concerning different methods for fatty acids derivatization are available in the literature [5, 7, 8]. On the basis of literature data presented by Larreta et al. [11], who applied derivatization of VFAs by the use of *N*-methyl-*N*-(*tert*-butyldimethylsilyl)trifluoroacetamide (MTBSTFA) and SPME method, it was decided to perform the same reaction by SDME technique. Application of VFAs derivatives (with MTBSTFA) characterized by a significantly higher molecular weights in the case of FID detector should result in considerably lower LODs of analytes.

Derivatization reactions can be performed prior to the step of extraction in the solution studied (in-sample derivatization), and simultaneous extraction and derivatization can be carried out in hanging drop (in-drop derivatization), in syringe needle (in-syringe derivatization), outside extraction system (in-microvial derivatization), and in particular cases in heated injection port of chromatograph (in-port derivatization) [14]. The preliminary studies taking advantage of both headspace and direct SDME methods showed that derivatization of acids did not proceed when drop of a suitable solvent containing MTBSTFA was placed in headspace or directly in water solution. It is probably caused by the fact that MTBSTFA is sensitive to moisture. The only possible method to perform derivatization by MTBSTFA was extraction outside the extraction system. It consists in introduction of drop together with the extracted VFAs into microvial and performing derivatization reaction in it.

**Fig. 3:**
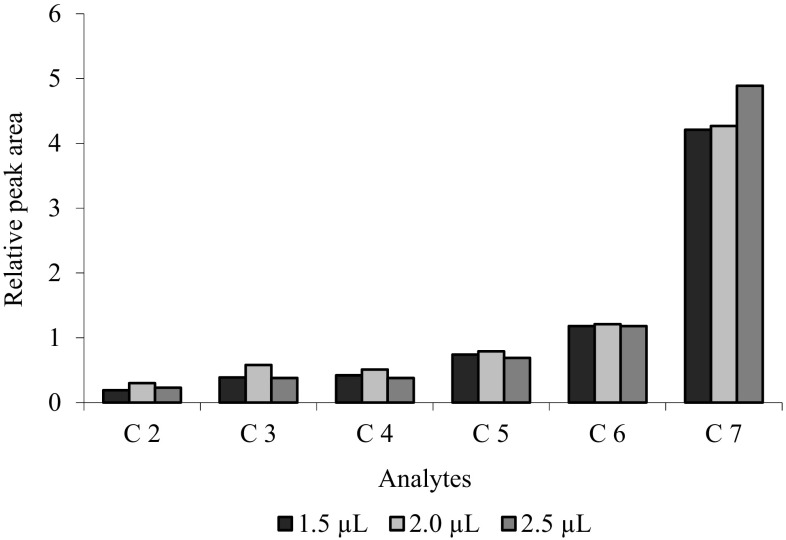
Effect of microdrop volume on the extraction efficiency for DI-SDME method. Concentration of analytes: 85–155 mg L^−1^, solvent: carbon tetrachloride, solution temperature: 22 °C, time extraction: 12 min, salt addition: 4% NaCl, and stirring rate 500 rpm

In the next stage, the optimum parameters were determined. The first step of studies comprised selection of conditions for chromatographic analysis (oven program) to obtain suitable separation of peaks, because as a result of reaction, chromatogram contained extra peaks derived from derivatization reagent, unreacted acids and new reaction products. By stipulating the temperature oven program, separation of all compounds was achieved and it was presented in Fig. 3b.

To achieve separation of chromatographic peaks, it was also necessary to select a suitable concentration of MTBSTFA; in such a manner that the peak derived from this compound did not eluate with analytes and simultaneously, the efficiency of derivatization was high.

After extraction, the drop was dislodged to microsyringe, and then, it was placed in microvial of 0.3 mL. Then, various amounts of MTBSTFA were introduced into microvials. It was necessary to dilute MTBSTFA in extractant (carbon tetrachloride with internal standard). The best effects were obtained by introducing 0.5 µL of MTBSTFA solution (diluted in the ratio 1:4) into 2 µL of extractant drop. After derivatization, solution was reintroduced into microsyringe and chromatographic analysis was performed. The derivatization reaction was performed at room temperature for different times (1–3 min). The experimental results showed that the derivatization could complete in a very short time of 1.0 min.

The repeatability and the calibration curves were studied under the optimized conditions. The results of studies on precision (RSD), the determined correlation coefficients (*r* ^2^), and the limits of detection are presented in Table 1.

A slightly lower *R* ^*2*^ values were obtained by applying DI-SDME method with derivatization in comparison with DI-SDME method exclusively. Higher *r* ^*2*^ values were obtained only in the case of butyric and heptanoic acids. Application of derivatization slightly reduced precision of analyses (max. RSD = 11.4% for caproic acid). Lower RSD values were obtained for heptanoic and acetic acids, and RSD were 9.0 and 7.2%, respectively. The highest limit of detection was obtained for acetic acid, i.e., on the level of 8.3 mg L^−1^; however, as the chain length was longer, LODs decreased to heptanoic acid for which LOD was 0.008 mg L^−1^. By comparing the LODs obtained by the developed DI-SDME-GC-FID method with the results obtained by Ábalos et al. [1] who applied HS-SPME-GC-FID method, it was found that for the first four acids, lower LODs were for HS-SPME-GC-FID method. Only for higher acids (caproic and heptanoic), the results for SDME method are comparable and LOD is lower even for heptanoic acid. The results of experiments suggest that DI-SDME method can be applied more successfully to less volatile acids. The enrichment factor (EF) was calculated as the ratio of the final concentration of acids in droplet after extraction to the initial concentration of acids in the aqueous solution. The results indicate that EFs are between 92 and 148 (Table 1).

Application of microdrop to the extraction of VFAs has one very important advantage, i.e., time of analysis by SDME method is considerably shorter in comparison with SPME. Larreta et al. [7] performed extraction by the use of 50/30/µm fibre DVB/CAR/PDMS and derivatization by MTBSTFA and time required to perform extraction was 99 min (exposure time of the fibre to the derivatization—60 min, extraction time—15 min, desorption time—9 min, and heating of fibre in feeder—15 min). Time of extraction performed by this method is only 13 min (extraction time—12 min and reaction time—1 min). Such a significant shortening of time was achieved by applying each time the fresh drop of solvent for extraction, what allowed to avoid time-consuming sorption of derivatization reagent and time-consuming desorption of analytes from fibre (so as in SPME).

## Conclusions

DI-SDME combined with MTBSFA derivatization is a rapid, sensitive, and reproducible method for analysis of VFAs in a water samples. High precision and simple sample preparation enable the use of this method for routine investigations in both the industrial and research laboratories. The proposed method is advantageous in many practical aspects, such as simplicity and short time of extraction and only 2 µL of solvent consumption.

The procedure developed is a good source for application in further VFAs analyses in different natural samples. It should be noticed that application of detectors other than FID could significantly increase sensitivity of analyses.
